# Diagnostic Challenges of Pneumocystis jirovecii Pneumonia in Methotrexate-Induced Pancytopenia: A Report on a Fatal Case and Review of the Literature

**DOI:** 10.7759/cureus.82542

**Published:** 2025-04-18

**Authors:** Takuma Ikeda, Kenta Iijima, Fuyuki Mukaizawa, Kensuke Fujiwara

**Affiliations:** 1 Department of Respiratory Medicine, Hyogo Prefectural Amagasaki General Medical Center, Amagasaki, JPN; 2 Department of Infectious Diseases, Hyogo Prefectural Amagasaki General Medical Center, Amagasaki, JPN; 3 Department of Hematology, Hyogo Prefectural Amagasaki General Medical Center, Amagasaki, JPN

**Keywords:** ciprofloxacin-induced pancytopenia, methotrexate, non-hiv pcp, pancytopenia, pneumocystis jirovecii, pneumocystis pneumonia (pcp)

## Abstract

Early detection and diagnosis of *Pneumocystis jirovecii* pneumonia (PCP) among non-HIV patients is crucial because of its rapid course. However, when PCP is suspected due to respiratory symptoms in pancytopenia patients, performing bronchoalveolar lavage is often challenging because of progressing respiratory failure and elevated risk of bleeding.

We report the case of a 78-year-old woman with rheumatoid arthritis who developed PCP during methotrexate (MTX)-induced pancytopenia. One week before hospital admission, she presented with fever and malaise without respiratory symptoms. We made a definitive diagnosis via Grocott-stained sputum with reference to her elevated plasma β-D-glucan. Respiratory failure was already advanced at diagnosis, and the patient died on hospital day 11.

Because MTX use and MTX-induced pancytopenia increase the risk of PCP, fever during pancytopenia may indicate the need for plasma β-D-glucan and high-resolution computed tomography for its early detection and treatment, even without respiratory symptoms. Minimally invasive techniques such as Grocott-stained sputum or real-time polymerase chain reaction (PCR) of sputum may be helpful in diagnosis when bronchoscopy is not feasible due to pancytopenia.

## Introduction

*Pneumocystis jirovecii* pneumonia among HIV patients (HIV PCP) progresses gradually, whereas non-HIV PCP advances rapidly within a few days to weeks [1]. While the mortality rate of HIV PCP is reported to be around 10%-20%, that of non-HIV PCP is significantly higher, ranging from 30% to 50% [2-4]. Low-dose administration of trimethoprim/sulfamethoxazole (TMP/SMX) can prevent fatal PCP. Therefore, prophylactic administration of TMP/SMX is necessary for high-risk patients, such as those receiving multiple immunosuppressive agents or more than 20 mg of prednisolone per day.

The difference in mortality between HIV and non-HIV PCP is attributed to host-related factors. Compared to HIV PCP, non-HIV PCP typically presents with a lower amount of *P. jirovecii* but shows a higher neutrophil count in bronchoalveolar lavage (BAL) fluid, which correlates with poor oxygenation and patient survival [5]. In non-HIV PCP, it is not the amount of *P. jirovecii* but lung inflammation that contributes to lung injury.

Given the rapid progression of non-HIV PCP, early diagnosis and initiation of treatment are important, as delays adversely affect patient outcomes [6]. However, establishing a definitive diagnosis poses difficulties, particularly in pancytopenia patients. BAL, a gold standard of the definitive diagnostic procedure for PCP, may be challenging to perform due to progressing respiratory failure and the elevated risk of airway bleeding associated with pancytopenia.

We report a fatal case of a patient diagnosed with PCP through the Grocott-stained sputum and real-time polymerase chain reaction (PCR) of sputum who presented with fever and malaise but without respiratory symptoms. This patient presented with pancytopenia, which was caused by impaired excretion of methotrexate (MTX), a folate metabolism antagonist, due to dehydration, hypoalbuminemia, and pleural effusion. Additionally, drawing on existing literature, we explore diagnostic strategies for the early detection of PCP in patients with MTX-induced pancytopenia.

## Case presentation

A 78-year-old woman with a four-year history of rheumatoid arthritis (RA) was brought to another hospital by ambulance after collapsing at home, accompanied by general malaise and fatigue. She had been treated with 4 mg of MTX weekly and 5 mg of prednisolone daily, but had not received folic acid or prophylactic TMP/SMX. Three weeks before collapsing, she had presented with fever and malaise. Despite mild pancytopenia observed during her regular outpatient visit two weeks before collapsing, she was kept under observation as her condition was otherwise stable.

**Figure 1:**
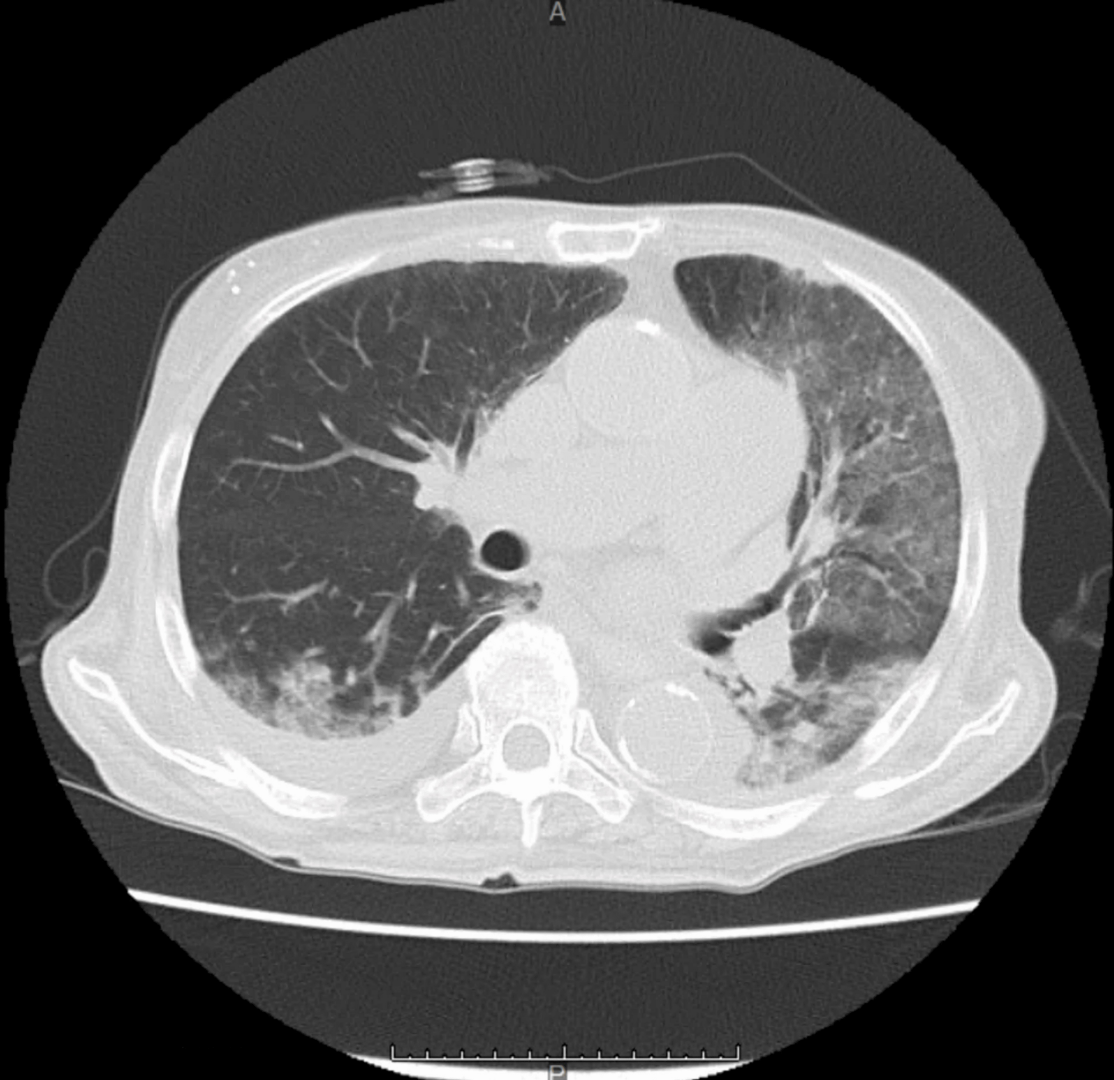
CT scan of the chest CT: computed tomography

On examination, her blood pressure was 101/67 mmHg, heart rate was 118 per minute, respiratory rate was 24 per minute, percutaneous oxygen saturation was 97% on ambient air, and body temperature was 37.1℃. She was alert and oriented. No cough or respiratory symptoms were noted. Laboratory data revealed pancytopenia. Unexpectedly, a chest computed tomography (CT) revealed ground-glass opacities in the left lung and bilateral dorsal consolidations (Figure 1).

She was admitted to the hospital for community-acquired pneumonia and treated with ampicillin/sulbactam. On the second hospital day, she developed septic shock, which resulted in an additional administration of tazobactam/piperacillin, noradrenaline, and prednisolone. By the sixth hospital day, she was successfully weaned off noradrenaline. However, her pancytopenia worsened, and plasma β-D-glucan levels elevated. She was transferred to our hospital for further evaluation and treatment. She was given 10 units of platelets before transfer.

After the transfer, her respiratory status worsened, requiring 2 L/minute of oxygen via nasal cannula. Pan-inspiratory crackles were auscultated in the left lower lung field, and purpura was observed on both upper and lower limbs. Table 1 shows the laboratory findings.

**Table 1:** Laboratory findings IFCC: International Federation of Clinical Chemistry, ND: not determined, PCO_2_: partial pressure of carbon dioxide, PO_2_: partial pressure of oxygen, HCO_3_: bicarbonate

Parameters	Patient values	Reference range
White blood cell count	0.5 × 10^9^	3.3-8.6 × 10^9^/μL
Neutrophils	76	43%-65%
Lymphocytes	23	20%-50%
Red blood cell count	2.65 × 10^12^	3.86-4.92 × 10^12^/μL
Hemoglobin	8.5	11.6-14.8 g/dL
Mean corpuscular volume	93.6	83.6-98.2 fL
Platelet count	52 × 10^9^	158-348 × 10^9^/µL
Total protein	5.4	6.6-8.1 g/dL
Albumin	2.7	4.1-5.1 g/dL
Total bilirubin	0.8	0.4-1.5 mg/dL
Aspartate aminotransferase	21	13-30 U/L
Alanine aminotransferase	21	7-23 U/L
Lactate dehydrogenase (IFCC)	405	124-222 U/L
Blood urea nitrogen	48.9	8-20 mg/dL
Creatinine	1.57	0.46-0.79 mg/dL
Sodium	138	138-145 mmol/L
Potassium	3.4	3.46-4.8 mmol/L
Chloride	107	101-108 mmol/L
Calcium	7.7	8.8-10.1 mg/dL
Folic acid	1.6	3.6-12.9 ng/mL
C-reactive protein	7.7	0.00-0.14 mg/dL
β-D-glucan	317	<11 pg/mL
Methotrexate	0.005	ND (μmol/L)
pH (arterial gas)	7.475	7.35-7.45
PCO_2_	25	32-43 Torr
PO_2_	64	69-116 Torr
HCO_3_	18	20-24 mmol/L
P. jirovecii real-time PCR	7 × 10^3^	<4.0 × 10 copy/μgDNA

Given the rapid deterioration of her respiratory condition, elevated plasma β-D-glucan levels, and bilateral ground-glass opacities on chest CT, we clinically diagnosed her with severe PCP. Treatment was initiated with intravenous TMP/SMX 480 mg/2,400 mg/day and intravenous prednisolone 80 mg/day. Due to the concurrent presence of pancytopenia, meropenem was also administered.

**Figure 2:**
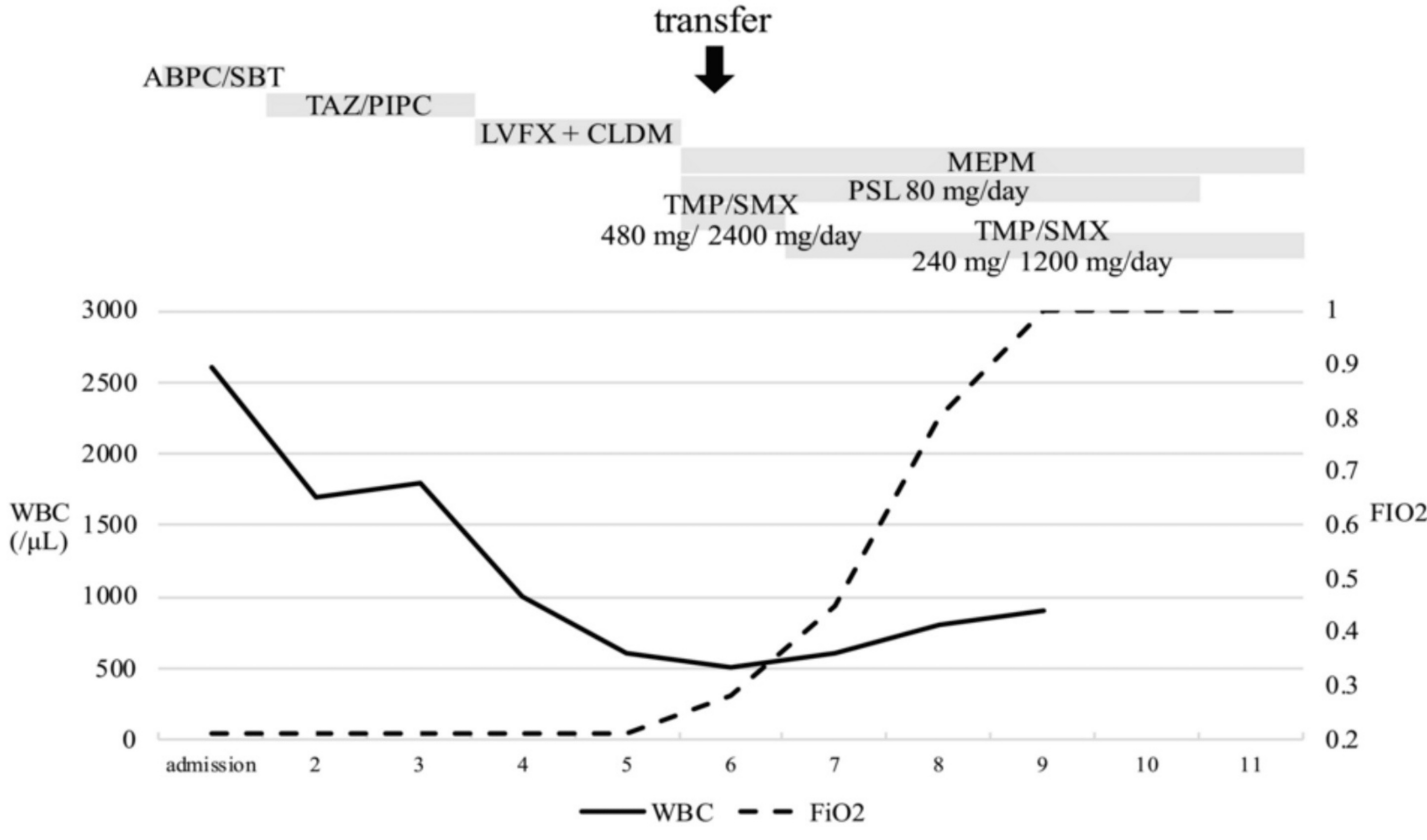
Clinical course ABPC/SBT: ampicillin sulbactam, TAZ/PIPC: tazobactam piperacillin, LVFX: levofloxacin, CLDM: clindamycin, MEPM: meropenem, PSL: prednisolone, TMP/SMX: trimethoprim/sulfamethoxazole, WBC: white blood cell, FiO_2_: fraction of inspiratory oxygen

On evaluation of pancytopenia, drug-induced pancytopenia was suspected. Both hemophagocytic syndrome and thrombotic microangiopathy were ruled out. Blood tests revealed folate deficiency and detectable serum MTX levels, even 11 days after the last MTX dose. Consequently, MTX-induced pancytopenia was diagnosed, and oral calcium folinate was administered. Despite aggressive treatment, her respiratory status worsened rapidly the following day. She died on the 11th hospital day (Figure 2).

A yeast-like fungus was detected in Grocott-stained sputum during hospitalization. Later, *P. jirovecii* was confirmed in the sputum through real-time PCR, confirming the diagnosis of PCP.

## Discussion

Diagnostic delays

This case is a fatal case of PCP that developed during MTX-induced pancytopenia. The initial symptoms presented were fever and malaise without accompanying respiratory symptoms. While a diagnosis of PCP was made later based on elevated plasma β-D-glucan levels and confirmed via Grocott-stained sputum, the patient succumbed to progressive respiratory failure, emphasizing the urgency of early detection and treatment. We believe the reasons for treatment failure were pancytopenia and a delay in the diagnosis and treatment of PCP. Mortality rates for PCP in non-HIV patients are high, ranging from 70% to 100%. However, when PCP is treated within the first three days of admission, the likelihood of survival exceeds 90% [6]. Early diagnosis and rapid initiation of treatment within this critical 72-hour window are crucial for improving outcomes.

One of the reasons for delayed diagnosis and treatment is that the initial symptoms of PCP in patients with MTX-induced pancytopenia often lack respiratory symptoms at onset. We searched PubMed for case reports of RA patients who developed PCP in MTX-induced pancytopenia between July and August 2023. The search formula is: (("Methotrexate"[Mesh] OR "Methotrexate"[Title/Abstract] OR "MTX"[Title/Abstract] OR "Amethopterin"[Title/Abstract])) AND (("Hematologic Diseases"[Mesh] OR "Hematologic Diseases"[Title/Abstract] OR "Pancytopenia"[Mesh] OR "Pancytopenia"[Title/Abstract] OR "Cytopenia"[Title/Abstract] OR "Thrombocytopenia"[Mesh] OR "Thrombocytopenia"[Title/Abstract] OR "Leukopenia"[Mesh] OR "Leukopenia"[Title/Abstract] OR "Neutropenia"[Mesh] OR "Neutropenia"[Title/Abstract] OR "Anemia"[Mesh] OR "Anemia"[Title/Abstract])) AND (("Pneumocystis Pneumonia"[Title/Abstract] OR "Pneumocystis Infections"[Mesh] OR "Pneumocystis Infections"[Title/Abstract] OR "Pneumocystis carinii"[Mesh] OR "Pneumocystis carinii"[Title/Abstract] OR "Pneumocystis jirovecii Pneumonia"[Title/Abstract] OR "PCP"[Title/Abstract])). Thirty-four articles were identified, of which 13 were case reports and reviews. Of these, nine cases were deemed relevant by two reviewers (T.I. and K.I.) and were limited to case reports of MTX-induced pancytopenia in patients with RA. According to our review of 10 cases, including ours (Table 2), half of the patients (5/10) had fever or malaise, and a few days or a week later, they presented with respiratory symptoms and hypoxia.

**Table 2:** Cases of pneumocystis pneumonia in MTX-induced pancytopenia MTX: methotrexate, TMP/SMX: trimethoprim sulfamethoxazole, ND: not determined, CT: computed tomography

Study	Age/sex	Symptoms	MTX (/week)	Date of hypoxia	Lymphocyte	Date of TMP/SMX	Dosage of TMP/SMX	Outcome
Lang et al. [7]	63/F	Two weeks of fever, a few days of malaise, cough, and dyspnea	5-15 mg	ND	1,080/μL	ND	2,600 mg/12,600 mg/day	Survived
Wollner et al. [8]	49/F	Fever, pancytopenia, recovery on day 4, fever on day 7, and shortness of breath	7.5-15 mg	ND	595/μL	ND	20 mg/100 mg/kg/day	Survived
	56/F	Malaise, cough, and faint	7.5 mg	ND	198 /μL	ND	20 mg/100 mg/kg/day	Survived
	64/F	Weakness, chills, night sweats, and cough	15 mg	ND	154 /μL	ND	20 mg/100 mg/kg/day	Dead
Stenger et al. [9]	63/M	Fever, malaise, cough, and weight loss	5-7.5 mg	ND	450/μL	ND	Cotrimoxazol 4 × 1,440 mg/day	Dead
Kitsuwa et al. [10]	62/M	Fever, pancytopenia, pneumonia on day 3, recovery on day 5, fever on day 9, and hypoxia	7.5 mg	Day 9 (after fever)	480/μL	Day 10	720 mg/3,600 mg/day	Dead
Arakawa et al. [11]	94/F	Three days of fever, cough, and pancytopenia	6 mg	Day 6	164/μL	Day 8	320 mg/1,600 mg/day	Survived
Shima et al. [12]	68/F	Pancytopenia and abnormal shadow on CT	6 mg	Day 1	506/μL	Day 3	ND	Survived
Doig et al. [13]	62/M	Six weeks of lethargy, night sweats, and mouth ulcers; fall on day 1; and fever and cough on day 3	20 mg	Day 4	500/μL	Day 9	960 mg/4,800 mg/day	Survived
Present case	78/F	Three weeks of fever and malaise; fall on day 1; septic shock on day 2; and recovery on day 6	4 mg	Day 6	104/μL	Day 6	480 mg/2,400 mg/day	Dead

This discrepancy in symptom presentation is not unique to MTX-induced pancytopenia cases. In general, non-HIV PCP patients are less symptomatic at diagnosis compared to HIV-positive individuals. Specifically, respiratory symptom frequencies such as dyspnea (64% vs. 70%) and cough (51% vs. 69%) were significantly lower in non-HIV cases [14]. These statistics support the notion that in the absence of respiratory symptoms, a high index of suspicion for PCP should still be maintained in non-HIV patients, including conditions of MTX-induced pancytopenia.

Utility of sputum

When clinicians suspect PCP in non-HIV patients with subtle symptoms, diagnostic tests should be performed to enable early treatments that may improve prognosis substantially. In this context, plasma β-D-glucan is a reliable first-line diagnostic tool due to its higher sensitivity. The positive predictive value of *P. jirovecii* PCR is low at 51.5%, indicating the possibility of colonization [15,16]. However, it can be diagnostically meaningful in combination with plasma β-D-glucan and CT findings. Grocott stain positivity for PCP in the bronchoalveolar lavage fluid is low at 4.3%, and this is associated with a low amount of *P. jirovecii* in non-HIV PCP [17]. However, Grocott-stained sputum remains helpful and offers the advantage of being less invasive compared to BAL, especially in cases of pancytopenia, where the risk of bleeding due to thrombocytopenia further complicates matters.

Additionally, the sensitivity of these sputum examinations may be improved in the cases of pancytopenia, as in the present case, because leucopenia leads to decreased phagocytosis and a high amount of *P. jirovecii*. Further research is needed to determine which tests should be prioritized for the early diagnosis of patients with pancytopenia, including cost-effectiveness: plasma β-D-glucan, sputum analysis, BAL, and high-resolution chest CT.

Limitation

In this literature review, it is essential to consider the influence of publication bias. Severe adverse events and rare complications tend to be reported more frequently, whereas mild cases or those that recover with early intervention are less likely to be published. As a result, the incidence and prognosis may appear more severe than they actually are. The impact of concomitant medications or other underlying conditions is often not adequately examined. It is important to recognize the bias in reported cases and interpret the findings with caution.

## Conclusions

We encountered a case of PCP in MTX-induced pancytopenia. Reviews of past literature show that in approximately half of the cases, fever and general fatigue preceded the onset of respiratory symptoms by about one week. For patients with MTX-induced pancytopenia, clinicians should consider the possibility of PCP at the onset of non-specific symptoms like fever and malaise, even before respiratory symptoms manifest.

With regard to testing, we diagnosed PCP using Grocott-stained sputum, as bronchoscopy could not be performed due to respiratory failure. A remarkably high fungal load in the sputum was observed, which is presumed to have been triggered by leukopenia. In addition to plasma β-D-glucan, Grocott-stained sputum and PCR can be effective and less invasive diagnostic tools than BAL in pancytopenia patients to initiate prompt treatment.
